# Using caloric efficiency to estimate the net energy value of expelled, extruded soybean meal relative to dehulled, solvent-extracted soybean meal and its effects on growth performance of nursery pigs^1^^,^^2^

**DOI:** 10.1093/tas/txac003

**Published:** 2022-01-15

**Authors:** Allison K Blomme, Haley K Wecker, Mike D Tokach, Jason C Woodworth, Charles R Stark, Chad B Paulk

**Affiliations:** 1 Department of Grain Science and Industry, College of Agriculture, Kansas State University, Manhattan, KS 66506, USA; 2 Department of Animal Science and Industry, College of Agriculture, Kansas State University, Manhattan, KS 66506, USA

**Keywords:** caloric efficiency, expeller-extracted soybean meal, metabolizable energy, nursery pigs, soybean meal, solvent-extracted soybean meal

## Abstract

This study aimed to estimate the net energy (NE) value of expelled, extruded soybean meal (MSBM) relative to dehulled, solvent-extracted soybean meal (SSBM) and determine its effects on growth performance of late nursery pigs. A total of 297 pigs (DNA 241 × 600) were weaned (BW 5.10 kg) and placed into 60 pens (2 rooms of 30 pens) with 5 pigs per pen balanced by gender and weaning weight. Pigs were fed a common diet for 21 d. Then, pens of pigs (BW 9.3 kg) were randomly assigned to one of five treatments to provide 12 replications per treatment. Treatments consisted of increasing amounts of MSBM replacing SSBM in the diet (0%, 25%, 50%, 75%, and 100%). All diets were fed for 28 d and were formulated to 1.30% standardized ileal digestible lysine and met or exceeded requirements for amino acids, calcium, and phosphorus. The SSBM diet was formulated to 2,421 kcal/kg and NE was not balanced between diets. Analyzed values for CP, EE, CF, and total lysine for the SSBM were 47.28%, 0.47%, 3.80%, and 3.00%, whereas the MSBM contained 47.41%, 6.88%, 5.32%, and 2.99%, respectively. The MSBM had increased values for KOH solubility and trypsin inhibitor (83.62% and 7,026 TIU/g) compared to the SSBM (73.05% and 3,011 TIU/g), whereas urease activity was similar between the two (0.03 and 0.02 Δ pH, respectively). Data were analyzed using Proc GLIMMIX (SAS 9.4; Cary, NC) with pen as the experimental unit and room as the blocking factor. There was no evidence of differences in ADG and ADFI in pigs fed diets with increasing concentrations of MSBM. Pigs fed diets with increasing concentrations of MSBM had improved (linear, *P *< 0.001) G:F and caloric efficiency on an NE basis. Using caloric efficiency to estimate NE of the MSBM relative to SSBM, MSBM was estimated to have a value of 2,566 kcal/kg. In conclusion, MSBM contains approximately 123% of the energy of SSBM, which improved feed efficiency when fed to nursery pigs.

## INTRODUCTION

Oil is extracted from soybeans using either solvent or mechanical extraction. Soybean meal is a byproduct of the soy oil extraction process. Soy oil is primarily extracted with the use of a hexane mixture running through soybean flakes that had previously been cracked and dehulled (Andersen, 2011). This process is capable of removing up to 97.5% of the oil contained in soybeans, leaving as little as 0.5% of oil in the remaining meal (Andersen, 2011). Because of the efficiency of oil separation, the solvent extraction process is commonly used in soybean crush facilities. For the mechanical extraction process, the oil is extracted using an expeller press. The expeller presses the flaked soybeans through a horizontal shaft using a screw. As the flakes travel through the expeller, their compression increases because the volume of the shaft decreases. Oil is allowed to escape from the expeller while the resulting soybean meal remains. The expeller process leaves about 5% to 8% oil content in the mechanical extracted soybean meal (Boeck, 2011).

Solvent extracted soybean meal (SSBM) is commonly used in swine diets to meet protein and amino acid requirements, with pigs consuming about 24% of the soybean meal used to feed livestock (Stein et al., 2008; United Soybean Board, 2018). Mechanical extracted or expelled soybean meal (MSBM) typically has decreased concentrations of essential AA compared to SSBM but have been demonstrated to have improved standardized ileal digestibility coefficients. In addition, MSBM contains more oil which provides increased energy content of the diet (Woodworth et al., 2001; NRC, 2012). Differing soybean sources and processing can lead to intermediate fiber, fat, and energy content between NRC (2012) reported values for MSBM and SSBM. Therefore, it is necessary to accurately quantify the energy content of MSBM for use in swine diets. The objective of this study was to determine differences in growth performance of pigs fed increasing amounts of two different sources of soybean meal (SSBM and MSBM), by using changes in caloric efficiency (CE) to estimate MSBM NE value.

## MATERIALS AND METHODS

The protocol used in this study was approved by the Kansas State University Institutional Animal Care and Use Committee.

### Diets and Experimental Design

A total of five dietary treatments consisted of increasing amounts of MSBM (0%, 25%, 50%, 75%, and 100%) used to replace SSBM on a weight/weight basis. The SSBM was sourced local to Manhattan, KS from MKC (Moundridge, KS). The MSBM was sourced from a proprietary processing plant in Nebraska and was produced using high shear dry extrusion-pressing. The soybeans were dehulled to produce SSBM, but non-dehulled soybeans were used to produce MSBM. Representative samples of SSBM and MSBM were submitted to the Agricultural Experimental Station Chemical Laboratories (University of Missouri–Columbia, Columbia, MO) to be analyzed for available lysine (method 975.44, AOAC, 2012), KOH solubility (Parsons et al., 1991), trypsin inhibitor activity (method 22-40, AACC, 2006), and urease activity (method 22-90.01, AACC, 2006; Table 1). Also, representative samples of corn, SSBM, and MSBM were submitted for determination of total AA content (method 982.30; AOAC, 2012). Corn, SSBM, and MSBM were also analyzed (Agricultural Experimental Station Chemical Laboratories, University of Missouri–Columbia, Columbia, MO) for dry matter (method 934.01; AOAC, 2012), crude protein (method 990.03; AOAC, 2012), crude fiber (method 978.10, AOAC, 2012), ether extract (method 920.39; AOAC, 2012), calcium (method 985.01; AOAC, 2012), and phosphorus (method 985.01; AOAC, 2012).

**Table 1.** Proximate analysis and total amino acid content of corn, solvent-extracted soybean meal (SSBM), and expelled soybean meal (MSBM), as-fed basis^a^

Item, %	Corn	SSBM	MSBM
Dry matter	86.56	90.01	93.55
Crude protein	8.10	47.28	47.41
Crude fiber	1.78	3.80	5.32
Ether extract	2.54	0.47	6.88
Calcium	0.01	0.45	0.34
Phosphorus	0.26	0.67	0.64
KOH solubility	–	73.05	83.62
Trypsin inhibitor, TIU/g	–	3011	7026
Urease, Δ pH	–	0.02	0.03
Amino acids			
Alanine	0.55	2.00	1.99
Arginine	0.37	3.36	3.46
Aspartic acid	0.53	5.28	5.41
Cysteine	0.17	0.68	0.74
Glutamic acid	1.48	8.56	8.70
Glycine	0.32	1.97	2.01
Histidine	0.22	1.24	1.25
Isoleucine	0.30	2.31	2.33
Leucine	0.87	3.62	3.63
Lysine	0.27	2.99	3.05
Available lysine	–	2.93	2.96
Methionine	0.16	0.65	0.64
Phenylalanine	0.38	2.43	2.49
Proline	0.65	2.34	2.40
Serine	0.35	1.91	2.12
Threonine	0.27	1.78	1.78
Tryptophan	0.06	0.67	0.69
Tyrosine	0.18	1.67	1.69
Valine	0.38	2.38	2.36

A representative sample of each ingredient was obtained, homogenized, and submitted to the Agricultural Experimental Station Chemical Laboratories (University of Missouri-Columbia, Columbia, MO) for proximate and amino acid analysis.

A total of 300 pigs (DNA 241 × 600, Columbus, NE) were weaned at 21 d of age with an average body weight of 5.10 kg at the Kansas State University Swine Teaching and Research Center (Manhattan, KS). Pigs were weighed and assigned to pens (1.22 × 1.52 m) with five pigs/pen balanced by gender and body weight. Forty of the pens contained three gilts and two barrows and twenty contained three barrows and two gilts. All pigs were fed a common diet for 21 d until they weighed an average of 9.39 kg. During this transition phase, three pigs were removed due to illness leaving 297 pigs left on test. After the 21-d common diet, pens of pigs were randomly assigned to one of five dietary treatments balanced by gender, pen weight, and location within the barn for a total of 60 treatment pens and 12 replications per treatment. Pens contained a three-hole dry self-feeder and one nipple waterer to provide ad libitum access to feed and water.

The five dietary treatments were fed for 28 d and consisted of increasing amounts of MSBM (0%, 25%, 50%, 75%, and 100%) used to replace SSBM on a weight/weight basis. Diets were formulated based on NRC values for AA and SID coefficients for the SSBM, MSBM, and corn. Inclusion of feed-grade AA was used to balance AA between treatments. Diets were formulated to exceed the NRC (2012) requirement estimates for AA, Ca, and P and were not balanced for NE. The NRC (2012) values for NE were used for SSBM (2,087 kcal/kg, soybean meal, dehulled, solvent extracted) and corn (2,672 kcal/kg) for calculated NE in Table 2. Diets were provided in mash form. Feed disappearance and pen weights were recorded each week and were used to determine ADG, average daily feed intake (ADFI), gain to feed ratio (G:F), and CE. The actual energy value of MSBM relative to SSBM was estimated based on caloric efficiency (CE), which was obtained by multiplying ADFI by kcal of NE per kg of 0% diet (2,421 kcal/kg) and dividing by ADG for each treatment. Calculating the CE of the MSBM diet against the SSBM was selected because the MSBM was intermediate in profile between NRC (2012) reported analysis for dehulled, extruded-expelled soybean meal and extruded-expelled soybean meal. To obtain an energy estimate, the energy value of MSBM was adjusted for the slope of CE to be zero.

**Table 2.** Ingredient composition of experimental diets, as-fed basis

	MSBM replacement of SSBM^a^, %				
	0	25	50	75	100
Ingredient, %					
Corn	63.72	63.74	63.75	63.76	63.80
Soybean meal, dehulled solvent extracted	32.50	24.37	16.25	8.13	–
Soybean meal, expelled	–	8.13	16.25	24.38	32.50
Calcium carbonate	0.95	0.95	0.95	0.95	0.95
Monocalcium phosphate, 21.5 % P	0.95	0.95	0.95	0.95	0.95
Sodium chloride	0.35	0.35	0.35	0.35	0.35
l-lysine-HCl	0.48	0.47	0.46	0.46	0.45
dl-methionine	0.24	0.23	0.21	0.19	0.17
l-threonine	0.22	0.22	0.23	0.23	0.22
l-tryptophan	0.04	0.04	0.05	0.05	0.06
l-valine	0.12	0.12	0.12	0.12	0.12
Trace mineral premix^b^	0.15	0.15	0.15	0.15	0.15
Vitamin premix^c^	0.25	0.25	0.25	0.25	0.25
Phytase^d^	0.03	0.03	0.03	0.03	0.03
Total	100	100	100	100	100
Calculated analysis, %					
SID amino acids, %					
Lys	1.35	1.35	1.35	1.35	1.35
Ile:Lys	57	56	56	56	56
Leu:Lys	116	118	120	122	124
Met:Lys	39	38	38	37	36
Met + Cys:Lys	60	60	60	60	60
Thr:Lys	65	65	65	65	65
Trp:Lys	19.5	19.5	19.5	19.5	19.5
Val:Lys	70	70	70	70	70
His:Lys	37	38	38	39	40
Net energy, kcal/kg	2421	–	–	–	–
Crude protein, %	21.6	21.4	21.2	21.0	20.8
Crude fiber, %	2.5	2.5	2.4	2.4	2.3
Ca, %	0.72	0.72	0.72	0.72	0.72
STTD P, %	0.46	0.46	0.46	0.46	0.46
Analyzed values, %					
Dry matter	88.36	88.76	89.09	89.40	89.83
Crude protein	21.95	22.00	22.23	21.78	21.48
Crude fiber	2.48	2.38	2.57	2.82	2.45
Ether extract	1.39	1.50	1.98	2.62	3.05

MSBM, mechanical-extracted soybean meal; SSBM, solvent-extracted soybean meal.

Each kilogram contains 22 g Mn, 73 g Fe, 73 g Zn, 11 g Cu, 198 mg I, and 198 mg Se.

Premix contains a minimum of Vitamin A 1,653,439 IU/kg, Vitamin D3 661,376 IU/kg, Vitamin E 17,637 IU/kg, Vitamin B12 13.3 mg/kg, menadione 1,323 mg/kg, riboflavin 3,307 mg/kg, d-Pantothenic acid 11,023 mg/kg, niacin 19,841 mg/kg.

Ronozyme HiPhos (GT) 2700, DSM Nutritional Products, Parsippany, NJ.

### Chemical Analysis

Representative diet samples were obtained from each treatment and stored at −20 °C until analysis. Samples were analyzed for dry matter (method 934.01; AOAC, 2012), crude protein (method 990.03; AOAC, 2012), crude fiber (method 978.10; AOAC, 2012), and ether extract (method 920.39; AOAC, 2012) at Experiment Station Chemical Laboratories (Columbia, MO).

### Statistical Analysis

Data were analyzed as a generalized randomized complete block design with pen as the experimental unit and barn/room as the blocking factor using the PROC-GLIMMIX procedure of SAS (SAS Institute, Inc., Cary, NC). Single degree-of-freedom contrasts were constructed to test the linear and quadratic effects of increasing MSBM. Results were considered significant at *P* ≤ 0.05 and marginally significant at *P* ≤ 0.10.

## RESULTS AND DISCUSSION

### Chemical Analysis

The analyzed values for corn were similar to expected values based on NRC (2012) (Table 1). The SSBM had decreased ether extract (0.47%) compared to the expected NRC (2012) value (1.52%). Analyzed values for the MSBM were similar to reported values for dry matter, crude fiber, Ca, and P compared to expelled soybean meal values reported in NRC (2012). However, the MSBM had increased concentrations of EE, CP, and AA compared to reported values (NRC, 2012). Although the MSBM used in this experiment was not dehulled prior to expelling, the analyzed values for EE, CP, and AA were similar to soybean meal, dehulled, expelled (NRC, 2012); however, the analyzed values for MSBM still had increased crude fiber (5.32% vs. 3.30%), CP, and AA values. The analyzed crude protein and amino acids concentrations were similar between the SSBM and MSBM. The MSBM had a higher DM content than the SSBM by three percentage points. This increase in DM allowed for the MSBM to have a higher fiber value (retained hulls) but still have a similar nutrient profile to the SSBM for several nutrients. Baker and Stein (2009) observed increased ADF and NDF concentrations in extruded-expelled soybean meal as compared to solvent-extracted soybean meal as well. Trypsin inhibitor activity, KOH solubility, and urease activity were all lower for SSBM (3,011 TIU/g, 73.05%, and 0.02 Δ pH, respectively) than in MSBM (7,026 TIU/g, 83.62%, and 0.03 Δ pH, respectively). Although the trypsin inhibitor activity was greater in the MSBM compared to the SSBM, the analyzed activities were similar to those observed by Chen et al. (2020). It has also been established that soybean meals with a KOH between 73% and 88% and urease levels between 0.1 and 0.3 delta pH are considered acceptable quality (U.S. Soybean Export Council, 2006). Therefore, quality measurements for both SSBM and MSBM were within acceptable ranges.

### Growth Performance and NE Estimate

There was no evidence of difference in ADG or ADFI between the diets (Table 3). However, increasing MSBM concentration of the diet improved (linear, *P *< 0.001) G:F and NE CE of pigs. Experimental diets were formulated to have similar concentrations or above the recommend concentration of standard ileal digestible AA, Ca, and standardized total tract digestible P. Therefore, this improvement in G:F was likely caused by an increase in NE provided by MSBM. These differences in NE can be attributed to the increase in EE values for MSBM. As expected, this indicates that more oil was retained during the expelling process compared to the solvent extraction process (Andersen, 2011; Boeck, 2011). Woodworth et al (2001) determined that pigs fed diets containing dry extruded-expelled soybean meal or solvent-extracted soybean meal had similar growth performance when diets were formulated to be balanced for apparent ileal digestible lysine and metabolizable energy. The authors determined that dry extruded-expelled soybean meal with hulls and without hulls had 470 and 550 kcal/kg more ME than solvent-extracted without hulls (Woodworth et al., 2001). In the experiment reported herein, CE or “productive energy” was used to estimate the NE of a feed ingredient based on the known value of another (Graham et al., 2014; Gonçalves et al., 2016). The NE system was developed to determine the energy in an ingredient that is available for a pig to use for maintenance and production. The CE or “productive energy” used herein is the energy used for growth or protein deposition. Nursery pigs have potential to deposit body protein at a high rate, resulting in extra body protein gain and associated BW gain under any additional supply of balanced proteins. However, lipid tissues of nursery pigs grow at relatively low rates contributing less to the BW gain (Zhang et al., 2020). Therefore, the different EE content between MSBM and SSBM could potentially lead to risk in using the CE approach to precisely estimate the NE value of the two ingredients in nursery pigs. Although this method does not entirely capture the energy used between lipid deposition and protein deposition in nursery pigs, it has been used previously to understand the differences in energy content of two ingredients in pigs (Cemin et al., 2020). Calculating the NE content of the MSBM based on CE, estimates that MSBM (2,566 kcal/kg) reported herein are 479 kcal/kg greater than the NRC NE value of SSBM (2,087 kcal/kg). This is similar to results observed by Velayudhan et al (2015) who found that dry extruded-expelled soybean meal had an average NE of 2,544 kcal/kg. Interestingly, this value is more similar to the NRC (2012) reported value for dehulled, expelled soybean meal (2,598 kcal/kg) than the expelled soybean meal (2,344 kcal/kg). The greater caloric value for the MSBM may be due to the dry matter difference between the MSBM (93.55%) and the SSBM (89.98%), but this would only account for 55 kcal/kg when the two soybean meals are evaluated on a dry matter basis. Although the MSBM contained a greater EE than the SSBM, the MSBM also contained more crude fiber. As a result, the amount of energy that could be attributed to EE content is unable to be quantified with the current method. Additional research also determined similar NE values with extruded-expelled soybean meal (2,552 kcal/kg) providing 494 kcal/kg of additional NE as compared to solvent extracted soybean meal (2,028 kcal/kg; Rodriguez et al., 2020).

**Table 3.** Effects of increasing expelled soybean meal replacing solvent-extracted soybean meal on growth performance and caloric efficiency of pigs^a^

	Treatment, %MSBM^b^						Probability, *P < *	
	0	25	50	75	100	SEM	Linear	Quadratic
BW, kg								
D 0	9.4	9.4	9.3	9.3	9.3	0.20	0.899	0.882
D 28	26.4	26.6	27.3	26.8	26.6	0.54	0.672	0.335
D 0 to 28								
ADG, g	602	604	609	622	618	0.01	0.249	0.908
ADFI, g	903	888	896	883	874	0.02	0.315	0.883
G:F, g/g	666	681	680	705	708	0.01	<0.001	0.934
CE^c^, kcal/kg	3,643	3,562	3,565	3,435	3,423	46.9	<0.001	0.966

A total of 297 pigs with an initial BW of 9.3 kg were used in a 28-d growth trial with 5 pigs per pen and 12 replicates per treatment.

MSBM, mechanical-extracted soybean meal.

CE, caloric efficiency obtained by multiplying ADFI by NE of 0% diet (2,421 kcal/kg) and dividing by ADG for each treatment.

## CONCLUSION

Extruded-expelled soybean meal used in the experiment conducted herein had similar concentrations of CP and essential AA and increased EE and crude fiber compared to the solvent-extracted soybean meal. Late nursery pigs fed increasing concentrations of extruded-expelled soybean meal did not demonstrate a difference in ADG or ADFI but did have improved G:F. Caloric efficiency was improved as the amount of extruded-expelled soybean meal increased. These results determined a NE estimate of 2,566 kcal/kg for extruded-expelled soybean meal, which is equivalent to 123% of the NE of solvent-extracted soybean meal.
